# Efficacy of web-based self-management interventions for depressive symptoms: a meta-analysis of randomized controlled trials

**DOI:** 10.1186/s12888-021-03396-8

**Published:** 2021-08-11

**Authors:** Yue Pang, Xin Zhang, Ruitong Gao, Linqi Xu, Meidi Shen, Hongyu Shi, Yuewei Li, Feng Li

**Affiliations:** School of Nursing, Jilin University, Changchun, China

**Keywords:** Depression, Web-based, Self-management, Meta-analysis

## Abstract

**Background:**

The incidence of depression is increasing worldwide. Depression can lead to poor physical health and even suicide. However, in high-income countries, only about 50% of the people with depression receive appropriate therapy, and the detection rate of depression in low- and middle-income countries is relatively lower. Web-based self-management enables remote treatment and solves the problem of insufficient psychological treatment resources. Many past studies have evaluated the effectiveness of web-based self-management of depression, but there has been no synthesis of evidence. Therefore, this study conducted a meta-analysis of the effectiveness of web-based self-management for depressive symptoms.

**Method:**

Six electronic databases (Cochrane Central Register of Controlled Trials, PubMed, Web of Science, Embase, CINAHL, and PsycINFO) were searched in September 2020. All literature referring to the effects of web-based self-management on depression were shortlisted by performing the medical subject headings (MeSH) search combined with a text word search.

**Results:**

A total of 18 eligible randomized controlled trials were identified, and the results from 3055 participants were consolidated. The web-based self-management group exhibited a greater reduction in depressive symptoms than the control group (g = − 0.46; 95% CI: 0.62,0.30), and there was no evidence of publication bias. Subgroup analysis revealed that patients with moderate-to-severe depression benefited from web-based self-management interventions. In terms of interventions, those based on cognitive behavioral therapy (CBT) were highly effective. We noted that the longer the intervention time, the better was the improvement in the status of depression. Furthermore, it was established that participants who communicated with therapists and showed greater adherence to the intervention experienced significant improvement in their symptoms. The results of the intervention group were better than those of the waiting-list, treatment-as-usual, and online psychoeducation groups.

**Conclusions:**

Web-based self-management is a promising therapy for depression. Future research should aim to refine these aspects of the intervention to achieve a beneficial impact.

**Supplementary Information:**

The online version contains supplementary material available at 10.1186/s12888-021-03396-8.

## Introduction

According to the World Health Organization’s World Mental Health Survey (WMH), the lifetime prevalence of severe depression varies widely across country, with 19.2% people in the United States, 21% in France, 17.9% in the Netherlands, 6.6% in Japan, and 18.4% in Brazil being afflicted by this condition [1]. On an average, the lifetime prevalence of depression was noted to be higher in high-income countries (14.6%) than in low- and middle-income countries (11.1%) [2]. The high incidence of depression means that it is common, with the frequency of almost one in five people experiencing a depressive episode at some point in their lives. Depressive symptoms occur in 1 in 10 patients on average in primary care settings [3].

In addition to aggravating personal suffering, depression has been linked to poor physical health, impaired social functioning, and even suicide. Hence, the illness imposes a great burden on both individuals and society, especially considering the stress of clinical psychotherapy [4]. The presence of depression significantly influences the prevalence, costs, and outcomes of several common comorbidities [5]. The results of a Hong Kong-based cohort study suggest that depressive symptoms are likely to be independent causes of suicide [6, 7].

Depression is treatable, and the current treatment consists mainly of antidepressant medications and psychotherapy. Despite a large number of high-quality research evidence implying that both the methods are effective, only about half of the people with depression receive proper treatment [8, 9]. Nearly 50% of the world’s population lives in countries with less than one psychiatrist per 100,000 individuals [10]. Obstacles are rampant in both treatments. First of all, drug treatment can lead to low compliance owing to the stigma attached to it, especially among young patients. Second, concerning psychotherapy, there is a dearth of trained mental health personnel and psychological treatment institutions, which are far from meeting the needs of the patients [11, 12]. To sum up, although both interventions are effective, their implementation rate is low. Therefore, interventions that are both effective and easy to implement are urgently needed.

In this scenario, web-based self-management of depression may be a promising solution. To simplify, self-management of depression indicates that an individual is an active self-manager. Moreover, with or without the support of healthcare professionals, this individual can actively treat his/her depression gradually on an everyday basis, by performing activities such as obtaining disease information, drug management, symptom management, lifestyle changes, and actively seeking social support and communication, thereby reducing the possibility of relapse and tending toward a healthier life [13]. A concept similar to self-management is self-help, and most of the currently known self-help approaches for treating depression involve repackaged CBT-based technologies that aim to treat the acute symptoms of depression. Self-management of depression involves proactively learning ways to manage the depression over a longer period, which can teach the patients more useful skills that can continue to work above and beyond the short-term relief that may be otherwise gained from the conventional self-help strategies. Self-help for depression can be seen as self-management with short-term effects; hence, this manuscript also included articles on self-help for depression in the meta-analysis.

To understand the advantages of self-management of depression by using the Internet, when compared with face-to-face or telephonic intervention, web-based self-management involves no restrictions on cost, personnel, or resources and can therefore improve the efficiency of intervention, necessitating less therapist time [14].

The existing randomized controlled trials have asserted that self-management provided through the internet can help improve the treatment for patients with moderate and severe depression [15–18]. This intervention has long-term effects and is sustainable for 3 years [19]. Research has indicated that web-based self-management interventions can also prevent the recurrence of depression [20]. However, certain studies have alluded that web-based, guided self-help interventions are less effective than the standard nursing control group [21, 22]. Another study has demonstrated that Internet-based problem-solving therapy is not more effective in reducing symptoms of depression than that by receiving an unguided self-help book during the waiting list period at outpatient mental health clinics [23]. The current consensus on whether web-based self-management is effective for alleviating depressive symptoms remains debatable and hence unclear. Previous meta-analyses have shown that self-guided internet-based cognitive behavioral therapy (iCBT) is effective in treating depressive symptoms [24]. This meta-analysis aimed to expand the theoretical scope of intervention by including studies based on CBT, as well as on other theories, such as problem-solving treatment (PST), positive psychotherapy (PPT), emotion-focused therapy, and interpersonal therapy (IPT). Moreover, this study not only analyzed the effectiveness of intervention from the theoretical perspective but also considered the influence of intervention time and guidance.

In summary, the effectiveness of web-based self-management interventions for depression is controversial, hence it makes sense to conduct this meta-analysis. This study aimed to assess the effectiveness of self-management through the internet in reducing depressive symptoms. The outcome variables include the changes in the depression scores from baseline to post-intervention, measured using any of the well-validated depression scales. We also attempted to use subgroup analysis to explore which aspects of web-based self-management intervention have a high or low curative effect on depressive symptoms. The results of this study are expected to provide a preliminary direction for the formulation of such interventions in the future. The findings are also likely to guide treatment selection and further research in this field.

## Methods

### Registration

This meta-analysis abided by the PRISMA statement [25, 26]. To eliminate the bias of the researchers, the search strategy, inclusion criteria, data extraction, and pre-planned subgroup analysis were strictly monitored by the PROSPERO system review through a registration agreement (CRD42020223172).

### Literature search strategies

We searched the following databases from the establishment of the database to September 21, 2020: Cochrane Central Register of Controlled Trials, PubMed, Web of Science, Embase, CINAHL, and PsycINFO. All literature related to the effects of web-based self-management interventions on patients with depression was obtained by using the medical subject headings (MeSH) search combined with a text word search. The specific search strategy is depicted in Additional file 1. The reviews on related topics were searched and analyzed, all the references in the relevant articles were manually searched, and the studies that met the inclusion criteria of this research were included. The literature search process was simultaneously carried out by two authors, and if any differences emerged, the third author resolved them. The search results were imported into Endnote, and some preliminary filtering was done by browsing the titles and abstracts.

### Eligibility criteria

#### Inclusion criteria

We only included randomized controlled trials published in the English language from the establishment of the database until September 21, 2020. The minimum intervention period for inclusion in this study was 4 weeks. The literature selection process involved three independent researchers who evaluated the quality of the articles and resolved any differences through discussion.

The participants included in this study were required to meet the following criteria: depression was diagnosed by a physician based on DSM-IV, ICD-10, or assessed by any well-validated depression scales (BDI-II, CES-D, MADRS-S, and PHQ-9); no cognitive impairment or any other mental illness. The purpose of this study was to examine the impact of web-based self-management interventions on the participants’ depressive symptoms. Hence, there were no restrictions on any other clinical or demographic characteristics of the eligible participants.

Only web-based self-management interventions were included in this study, such as the imparting of self-management skills through websites, programs, and interactive games. Self-management intervention should include the introduction of depression knowledge; treatment to improve behavior, cognition, and emotion; methods to prevent recurrence; methods to improve sleep and physical health, and the necessary knowledge of medication. In this study, both therapist-contacting and non-contacting studies during the intervention were included.

The control groups in this study included the waiting-list group, treatment-as-usual group, and an online psychoeducation group. The waiting list group did not receive care until the post-test data were collected from both the groups. The treatment-as-usual group was provided the usual mental health care, while the online psychoeducation group was provided an online psychological education about depression.

At least one primary or secondary outcome measure illustrating the depressive symptoms was used.

#### Exclusion criteria

1. Duplicate articles were excluded.
2. Meeting reports, guidelines, newspapers, and summaries were excluded.
3. Randomized controlled trials with no control group were excluded.
4. If the implementation of self-management was not clearly stated in the intervention, the research was excluded.

### Study selection and data extraction

After literature screening, two independent researchers extracted data from published reports, and they filled identical data extraction forms. In case of disagreement, the problem was solved through discussion or contact with the author.

Each article made use of the data extraction table to retrieve the following data:

1. Study information (investigator, year, country, sample size, average age of the participants, diagnostic information or relevant inclusion criteria, study duration, adherence to the interventions, dropout rate, and trial quality)
2. Intervention functions (website/application name, self-managed program content, and any other intervention components)
3. Detailed information on the control group.
4. Impact on depressive symptoms (the total depressive symptoms were scored before and after by using any clinically validated assessment scale)

### Statistical analyses

All the included studies were assessed for quality according to the bias risk tool mentioned in the Cochrane Intervention System Assessment Manual. The research quality was assessed in terms of seven aspects (random sequence generation, allocation concealment, blinding of participants and personnel, blinding of outcome data, incomplete outcome data, selective reporting, and other bias), and the risk of each type of prejudice was classified as “unclear,” “low,” or “high.” [27] The results were entered in Review Manager 5.3 software (Nordic Cochrane Center, Copenhagen, Denmark) for generating bias risk graphs and bias risk summaries.

In this study, the statistical software Stata version 16.0 (Stata Corporation, College Station, TX, USA) was used to analyze the impact of web-based self-management intervention on the participants’ depression status. The data were in the form of continuous variables, which were expressed as mean ± standard deviation. Q test and I^2^ statistics were used to test the heterogeneity. Significant differences in Q values indicated that the selected research papers were heterogeneous, and I^2^ values of 25.0, 50.0, and 75.0% indicated low medium, and high heterogeneity, respectively. Considering the sampling errors between and within groups, the fixed effect model was employed for meta-analysis of homogeneous research, while the random effect model was utilized for meta-analysis of heterogeneous research. Standardized mean difference (as Hedges’ g) and 95% confidence intervals (CI) were exploited to express the effectiveness of the self-management intervention in treating depression when compared with the control group. Furthermore, Egger’s test and trim-and-fill analysis were used to evaluate any publication bias. *p* < 0.05 (two-sided) was considered statistically significant.

A pre-planned subgroup analysis was conducted to examine whether the effects of web-based self-management interventions on depressive states were different from those of the waiting list, treatment-as-usual and online psychoeducation groups. In addition, a series of exploratory investigations of subgroups and meta-regression analysis were done to ascertain the factors that may influence the effectiveness of web-based self-management interventions, including the detailed information on the sample (e.g., participants’ depression severity) and intervention characteristics (e.g., the theory of intervention support, the duration of intervention, and presence of therapist guidance).

## Results

### Study selection

**Fig. 1:**
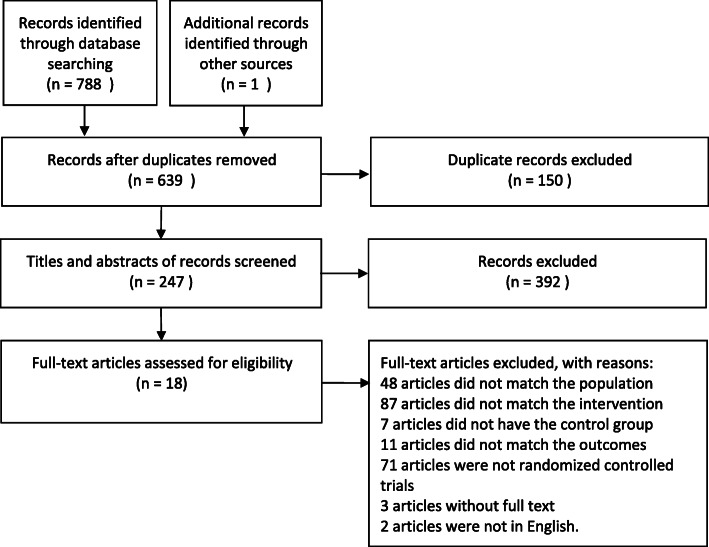
PRISMA Flow Chart of Study Selection

After careful screening of six databases, we obtained a total of 789 records. We eliminated 150 duplicate records and identified 639 relevant articles. In this process, by browsing the titles and abstracts, we excluded 392 articles that did not meet the inclusion criteria of this study and identified 247 articles for possible inclusion. Subsequently, by reading the full text, we excluded 48 articles that did not conform to depression, 87 articles that did not conform to intervention measures, seven articles that did not have a control group, 11 articles that did not conform to outcomes, 71 articles that were not randomized controlled trials, three articles for which full text could not be retrieved, and two articles that were not in English. Finally, we included 18 randomized controlled trials for analysis [15–18, 21–23, 28–38]. The article screening process is illustrated in Fig. 1.

### Study characteristics

Table 1 shows the details of each study. A total of 3055 participants were included from 18 studies published between 2005 and 2020. The adherence to the interventions and drop-out in each study are indicated in Table 1. Both the intervention and control groups comprised individuals with depression. Web-based self-management intervention was compared with the waiting list, treatment-as-usual, and online psychoeducation groups. The duration of the intervention ranged from 4 weeks to 12 weeks. The outcome measure was changed in depressive symptoms, which happened to be the primary outcome in 18 articles and the secondary outcome in one article. The Beck Depression Scale-II (BDI-II) was used in seven studies [15, 17, 28, 29, 31, 36, 38]. The Epidemiology Research Center Depression Scale (CES-D) was used in six studies [16, 21–23, 30, 33]. The Patient Health Questionnaire depression module (PHQ-9) was used in one study [35]. The Patient Health Questionnaire (PHQ-8) was used in one study [37]. The Hamilton Depression Scale (HRSD) was used in one study [18]. The Hospital Anxiety and Depression Scale (HADS) was used in one study [34]. The Montgomery-Eisenberg Depression Scale (MADRS-S) was used in one study [19]. The Modified Childhood Depression Rating Scale (CDRS-R) was used in one study [32].

**Table 1:** Details of Included Studies

Author/country/year	Sample type	Adherence to the interventions	N dropout(%)	Age (years,mean)	Intervention design	Control group	Treatment(T) & follow up(F)	Outcome (mean,SD)
Andersson, G.et al./Sweden/2005 [28]	Major depression (for the full CIDI) + mild-to-moderate depression (MADRS–S)	36/57 (63.16%)	117/32 (27.35%)	I:36.4 (11.5) C:36.3 (9.9)	The cognitive–behavioural self-help treatment: study on the website + the therapist gave an e-mail feedback + the discussion groups	A web-based discussion group	T:10w F:6 m	BDI: I: 12.2 (6.8) C: 19.5 (8.1)
Berger, T.et al./ Switzerland and Germany/2011 [29]	Moderate to severe depression (BDI-II)	25/25 (100%)	51/4 (7.84%)	38.8 (14.0)	The web-based self-help program (Deprexis) + scheduled e-mail contact with a therapist	Waiting list	T:10w F:6 m	BDI-II: I: 17.3 (10.2) C: 28.5 (9.4)
Boele, F. W.et al./Netherlands/2018 [21]	At least mild depressive symptoms (CES-D)	19/45 (42.22%)	89/36 (40.45%)	I:43.58 (11.69) C:46.43 (12.28)	Online guided self-help course	Waiting list	T:6w F:12 m	CES-D: I:18.84 (6.4) C:23.09 (7.1)
Bolier, L.et al./Netherlands/2013 [30]	mild to moderate depressive symptoms (CES-D)	95/143 (66.43%)	284/70 (24.65%)	43.2 (11.8)	An online self-help intervention (Psyfit): psycho-education + practical exercise	Waiting list	T:2 m F:6 m	CES-D: I:13.67 (6.69) C:15.39 (7.64)
Bücker, L.et al./Germany/2019 [31]	Self-reported depressive symptoms	47/64 (73.44%)	127/24 (18.90%)	I:44.02 (10.90) C:48.02 (10.95)	An internet-based self-help program (MOOD)	Care as usual (CAU)	T:6w	BDI-II: I:20.36 (14.70) C:18.68 (12.79)
Ebert, D. D.et al./Germany/2017 [16]	at least moderate symptoms of depression (CES-D)	129/130 (99.23%)	260/4 (0.02%)	50.8 (11.8)	A guided Internet-based self-help intervention (GET.ON Mood Enhancer Diabetes)	Treatment as usual + online psychoeducation about depression	T:2 m F:6 m	CES-D: I:21.1 (8.8) C:28.9 (8.7)
Eysenbach, Gunther et al./ Netherlands/2014	depressive symptoms (CES-D)	75/116 (64.66%)	231/60 (25.97%)	43.4 (9.2)	A worker-directed, Web-based, guided self-help intervention (Happy@Work)	Care as usual (CAU)	T:2 m F:12 m	CES-D: I:15.8 (10.6) C:18.3 (9.1)
Johansson, R.et al./Sweden/2012 [15]	Mild, moderate, and major depression (MADRS-S) + major depressive disorder (DSM-IV)	42/46 (91.30%)	92/4 (4.35%)	45.6 (14.0)	A guided self-help psychodynamic treatment (SUBGAP) + online support from a therapist	Non-directive online supportive treatment	T:10w F:10 m	BDI-II: I:11.48 (7.8) C:20.22 (7.8)
Kenter, Robin Maria Francisca et al./Netherlands/2016 [23]	major depressive disorder (DSM-IV)	94/136 (69.12%)	269/85 (31.60%)	38.0 (11.4)	An Internet intervention on problem solving therapy	A self-help book format without any form of guidance.	T:8w F:N	CES-D:I:27.0 (15.1) C:25.9 (14.9)
Merry, S. N.et al./ New Zealand/2012 [32]	Moderate to severe depression (PHQ-9)	85/94 (90.43%)	187/17 (9.09%)	I:15.55 (1.54) C:15.58 (1.66)	An interactive fantasy game (SPARX)	Treatment as usual	T:2 m F:3 m	CDRS-R: I:33.92 (11.19) C:35.07 (9.71)
Moritz, S./Germany/2012 [17]	depressive symptoms (BDI)	82/105 (78.10%)	210/40 (19.05%)	I:38.00 (10.76) C:39.13 (15.82)	An online self-help program for depression (Deprexis)	Waiting list	T:8w F:N	BDI: :20.51 (12.22) C:25.67 (11.65)
Reins, J. A.et al./Germany/2019 [18]	major depressive disorder (DSM-IV)	54/65 (83.08%)	131/22 (16.80%)	41.6 (10.8)	A guided internet-based cognitive behavioural therapy (GET.ON Mood Enhancer)	Online Psychoeducation on Depression	T:6w F:3 m	HRSD: :13.75 (7.52) C:16.47 (9.45)
Roepke, A. M.et al./the United States/2015 [33]	clinically significant depression (CES-D)	20/93 (21.51%)	186/130 (69.89%)	I:42.28 (12.56) C:40.27 (13.06)	An innovative smartphone and Internet-based game (SuperBetter)	Waiting list	T:4w F:6w	CES-D: I:23.55 (13.73) C:27.36 (10.63)
Sander, L. B.et al./Germany/2020 [34]	Mild, moderate, and major depression (PHQ-9)	102/149 (68.46%)	295/71 (24.07%)	52.8 (7.7)	A guided, web-based self-help intervention (eSano BackCare-DP) + automated motivational text messages + e-coaches give written feedback to answering queries.	Treatment as usual	T:9w F:12 m	HAM-D score: I: 5.63 (3.88) C: 7.24 (5.38)
van Luenen, S. et al./Netherlands/2018 [35]	mild to moderate depressive symptoms (PHQ-9)	75/97 (77.32%)	188/36 (19.15%)	46.30 (10.63)	An internet-based self-help intervention consisted of cognitive behavioural therapy	Waiting list	T:10w F:6 m	PHQ-9: I:6.73 (3.00) C:8.60 (3.12)
Vernmark, K. et al./Sweden/2010 [36]	mild to moderate depressive (MADRS-S) + major depression (DSM-IV)	24/27 (88.89%)	56/7 (12.50%)	36.82 (12.9)	An Internet guided self-help depression program: weekly modules + homework assignments	Waiting list	T:8w F:6 m	BDI: I: 12.3 (7.3) C: 16.6 (7.9)
Wilson, M.et al./the United States/2018 [37]	at least moderate depressive symptoms (PHQ-9)	22/27 (81.48%)	53/6 (11.32%)	I:45.7 (12.8) C:47.5 (12.9)	An internet-based, self-directed program for depressive symptoms: Videos + interactive activities + homework exercises	Treatment as usual	T:4w F:8w	PHQ-8: I:18.40 (5.28) C:20.74 (5.66)
Zwerenz, R.et al./Germany/2019 [38]	at least moderate depressive symptoms (BDI-II) + a clinical diagnosis of depression (ICD-10)	101/115 (87.83%)	229/31 (13.54%)	48 (9.79)	A Web-based self-help program (Deprexis) + multimodal inpatient psychodynamic psychotherapy	An internet platform provides basic information about depression	T:12w F:6 m	BDI-II: I:18.52 (10.78) C:24.75 (10.74)

Note. *BDI-II* Beck Depression Scale II, *CES-D* The Epidemiology Research Center Depression Scale, *PHQ-9* The Patient Health Questionnaire – depression module, *PHQ-8* The Patient Health Questionnaire, *HRSD/HAMD* The Hamilton Depression Scale, *HADS* Hospital Anxiety and Depression Scale, *MADRS-S* The Montgomery-Eisenberg Depression Scale, *CDRS-R* A modified childhood depression Rating Scale, *CIDI* Composite International Diagnostic Interview, *DSM-IV* Diagnostic and Statistical Manual of Mental Disorders, *RADS-2* The Reynolds adolescent depression scale-second edition, *QIDS* Quick Inventory of Depressive Symptomatology, *ICD-10* International Classification of Diseases tenth version

### Risk of bias within studies

**Fig. 2:**
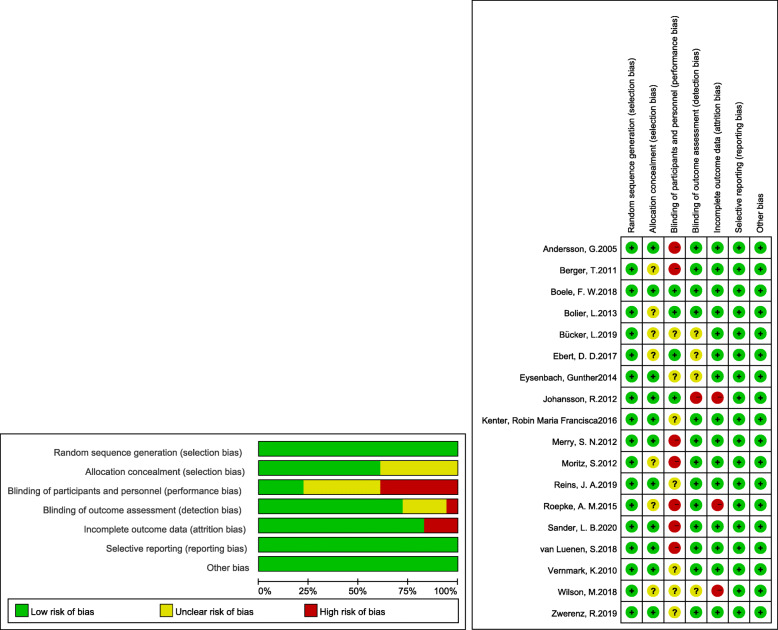
Quality Assessment of Randomized Controlled Trial: The Risk of Bias Graph and Summary. Note. +, low risk of bias;?, unclear risk of bias; −, high risk of bias

As portrayed in Fig. 2, we used the Cochrane risk bias assessment tool to ascertain the quality of the 18 randomized controlled trials. The random sequence generation risk of the articles was found below. Eleven studies [15, 18, 21–23, 28, 32, 34–36, 38] explicitly mentioned that allocation was hidden. The most common risk factor for bias in this meta-analysis was insufficient blindness on the part of the researchers or participants, with the researchers and participants not knowing the allocation of interventions in only four [15, 16, 21, 30] of the 18 studies. The measurement bias was not clear in four articles [16, 22, 31, 37], and one [15] was defined as high risk. Three of the articles [15, 33, 37] did not provide complete data. All the studies were low-risk in terms of selective reporting and other biases.

### The overall impact of web-based self-management interventions on depressive symptoms

**Fig. 3:**
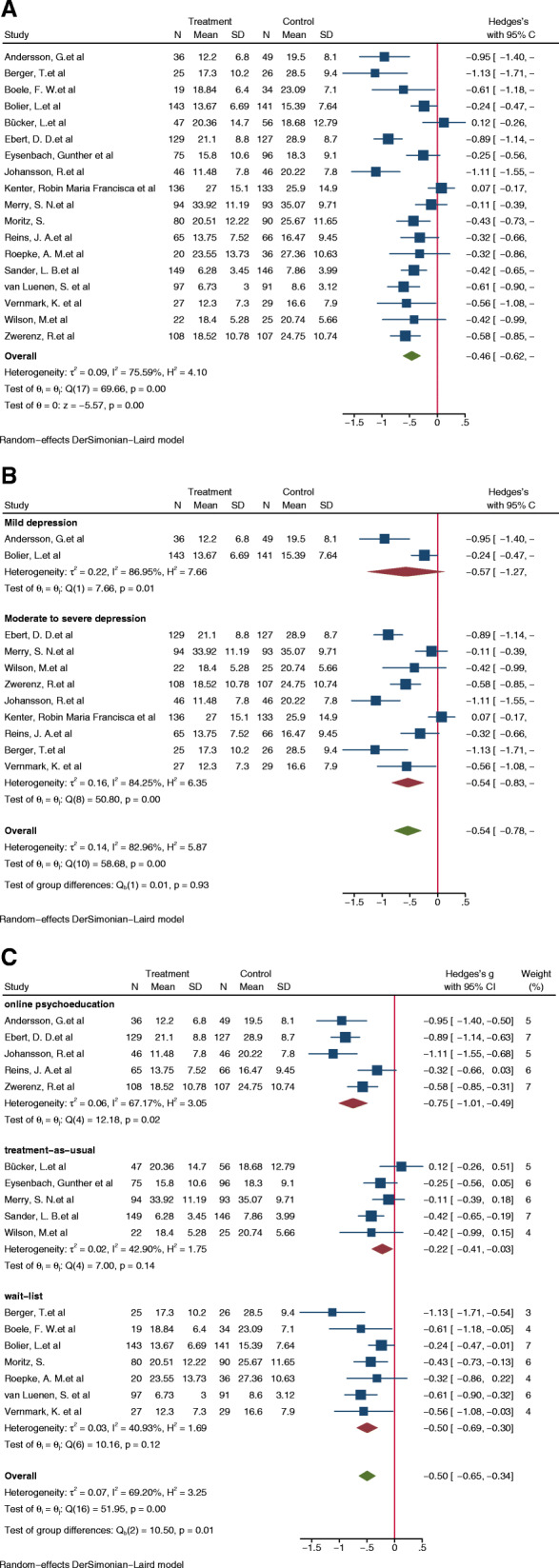
**A** Forest Plot of Web-based Self-management versus Control Group for Depression. **B** Subgroup Analysis: Effectiveness of Web-based Self-management in People with Different Levels of Depression. **C** Influence of Meta-analysis of Different Control Group

Figure 3 illustrates the combined effect of web-based self-management interventions on depressive symptoms as well as the individual effects of each study. The results from the random-effects model indicated that the web-based self-management intervention had a positive effect on the reduction of depressive symptoms when compared with the control group (*N* = 3055, g = − 0.46; 95% CI: − 0.62, − 0.30; *p* < 0.05). Despite the presence of some heterogeneity between the studies (I^2^ = 75.59%; *p* < 0.01), there was no evidence of publication bias (Egger test: P > |t| = 0.333 > 0.05). The funnel plots are presented in Additional file 3.

### Additional analysis

#### Population characteristics and effects on depressive symptoms

To determine the population in which web-based self-management interventions were most effective and to ascertain the sources of heterogeneity in this meta-analysis, we conducted a subgroup analysis based on the participants’ baseline depression severity (Fig. 3B). They were divided into mild depression groups and moderate-to-severe depression groups. The results implied that the combined effects on the moderate-to-severe depression group were statistically significant (g = − 0.54; 95% CI: − 0.83, − 0.24; *p* = 0.00); however, the combined effect on the mild depression group was not statistically significant (g = − 0.57; 95% CI: − 1.27, 0.13; *p* = 0.01). However, as the difference between the groups was not statistically significant, inter-group comparisons were not appropriate. Subgroup analysis indicated that the heterogeneity of this study may be due to the differences in the severity of depression at baseline (I^2^ = 82.96%; *p* = 0.00).

#### Intervention characteristics and effects on depressive symptoms

To further understand which aspects of the web-based self-management intervention were effective in tackling depression, we conducted subgroup analysis according to the different characteristics of intervention (such as the theory on which the intervention was based, the duration of intervention, and the contact of the therapist). The different characteristics of the interventions and the results of all subgroup comparisons are presented in Table 2 in a detailed manner. (The forest plot for subgroup analysis of the intervention is presented in Additional file 2).

**Table 2:** Post-hoc Analyses: Intervention Features

Aspects of intervention	Subgroup classification	Studies	Sample size (intervention/control)	Meta-analysis		Heterogeneity			Between groups tests	
				Hedges’ g	95% CI	Q	p	I^2^	Q	p
Intervention theory	CBT	9	617/645	−0.39	−0.58,-0.21	19.42	0.01	58.80%		
	CBT + other therapies	5	371/406	−0.44	−0.69,-0.19	10.38	0.03	61.47%		
	PST	3	284/294	−0.47	−1.17,0.24	29.64	0.00	93.25%	0.12	0.94
Intervention time	t ≤ 6w	5	173/217	−0.26	− 0.51,-0.02	5.68	0.22	29.62%		
	6w<t ≤ 8w	7	684/709	−0.33	−0.59.-0.08	33.37	0.00	82.02%		
	t > 8w	6	461/465	−0.73	−0.95,-0.50	12.69	0.03	60.61%	8.85	0.01
Guidance	Therapist guidance group	12	869/909	−0.60	−0.81,-0.38	50.93	0.00	78.40%		
	non-therapist guidance group	3	255/263	−0.17	−0.40,0.06	3.21	0.20	37.65%		
	Virtual health indicator guidance group	2	174/183	−0.27	−0.58,0.05	2.29	0.13	56.30%	7.49	0.02
Adherence to the interventions	≤ 90%	15	1118/1192	−0.35	−0.49,-0.21	36.26	0.00	61.97%		
	> 90%	3	200/199	−0.97	−1.17,-0.76	1.06	0.59	0.00%	23.60	0.00

Note. *CBT* cognitive behavioral therapy, *PST* problem-solving treatment

With regard to the different theoretical frameworks on which the intervention was based, the CBT alone group and the CBT combined with other theoretical frameworks group [such as CBT + PST, CBT + positive psychological therapy (PPT), CBT + IPT, and CBT + PPT + PST] demonstrated statistically significant improvements in depressive symptoms (CBT alone group: g = − 0.39; 95% CI: − 0.58, − 0.21; *p* = 0.01; CBT combined with other theoretical frameworks groups: g = − 0.44; 95% CI: − 0.69, − 0.19; *p* = 0.03). The group with PST alone did not display a statistically significant improvement in the depressive symptoms (g = − 0.47; 95% CI: − 1.17, 0.24; *p* = 0.00). Furthermore, the heterogeneity of the PST alone group was large (I^2^ = 93.25%). However, there was no significant difference among these 3 groups, making it unsuitable for comparison among the groups.

With regard to the intervention duration, the results implied the presence of significant differences in t ≤ 6w group, 6w < t ≤ 8w group, and t > 8w group (t ≤ 6w group: g = − 0.26; 95% CI: − 0.51, − 0.02; *p* = 0.22; 4w < t ≤ 8w group: g = − 0.33; 95% CI: − 0.59, − 0.08; *p* = 0.00; T > 8w group: g = − 0.73; 95% CI: − 0.95, − 0.50; *p* = 0.03). It was further discerned that the longer the intervention time, the higher the improvement in depressive symptoms. The differences among these subgroups were statistically significant (*p* = 0.01).

About the presence of guidance from a therapist, three groups were considered: the group communicating with the therapist, the group not communicating with the therapist, and the group communicating with the virtual healthcare provider. It was found that the group communicating with the therapist exhibited statistically significant improvement in depressive symptoms (g = − 0.60; 95% CI: − 0.81, − 0.38; *p* = 0.00), while the group not communicating with the therapist and the group communicating with the virtual healthcare provider did not exhibit a statistically significant improvement in depressive symptoms (g_1_ = − 0.17; 95% CI: − 0.40, 0.06; p_1_ = 0.20; g_2_ = − 0.27; 95% CI: − 0.58, 0.05; p_2_ = 0.13). The differences among these subgroups were statistically significant (*p* = 0.02).

Based on the extent of adherence to the intervention, we categorized the subjects into 2 groups: adherence ≤90% and adherence >90%. The results of subgroup analysis revealed that the intervention effect of high adherence group was better, and the difference between the groups was significant (g_1_ = − 0.35; 95% CI: − 0.49, − 0.21; g_2_ = − 0.97; 95% CI: − 1.17-0.76; *p* = 0.00).

#### The characteristics of the control group and effects on meta-analysis

Figure 3C depicts the influence of different control groups on the combined effect size. In our subgroup analysis, we identified that the web-based self-management intervention was statistically significant whether compared with the waiting list control group, the treatment-as-usual group or the online psychoeducation group (g_1_ = − 0.50; 95% CI: − 0.69, − 0.30; p_1_ = 0.12; g_2_ = − 0.22; 95% CI: − 0.41, − 0.03; p_2_ = 0.14; g_3_ = − 0.75; 95% CI: − 1.01, − 0.49; p_3_ = 0.02). The online psychoeducation control group showed medium heterogeneity (I^2^ = 67.17%), whereas the heterogeneity of the waiting list and treatment-as-usual control group was small (I^2^_1_ = 40.93%; I^2^_2_ = 42.90%). The separate types of control groups were comparable [Q (2) = 10.50, *p* = 0.01], and the control group was a significant moderator.

## Discussion

### Summary of evidence

To the best of our knowledge, this is the first meta-analysis exploring the impact of web-based self-management interventions on depressive symptoms. We systematically searched six databases and included 18 randomized controlled trials involving a total of 3055 participants.

Our analyses revealed that web-based self-management interventions exerted a positive effect on depressive symptoms, and there was no indication that publication bias might have influenced the results. We also conducted a subgroup analysis of the control group, and the results revealed that web-based self-management could significantly improve depression when compared with the waiting list, treatment-as-usual, or online psychoeducation control groups. The results of the different types of control groups were comparable, and the control group was a significant moderator. This result confirmed the effectiveness of web-based self-management for depression, as well as illustrated that the proposed approach is a potential intervention method.

A meta-analysis [39] has previously been published on the effects of self-management interventions in patients with depression; however, in this article, self-management was intervened by manual approaches. However, our study explored the effectiveness of self-management for depressive symptoms through the use of the Internet. Presently, discussion on which components of web-based self-management interventions play a role in improving depressive symptoms is lacking. However, with the rapid developments in the medical field, literature on psychological intervention via the internet or smartphones has increased dramatically. A meta-analysis [40] has been conducted on the influence of psychological intervention through smartphones on depressive symptoms, and the results asserted that the intervention had a considerable effect. This finding suggests that informational psychological intervention might be the future trend.

Subgroup analyses of the participant characteristics should be interpreted with caution. Considering the lack of significant difference between the mild and moderate-to-severe depression groups, it was impossible to compare between the groups. However, in the moderate-to-severe depression group, the symptoms of the participants were significantly improved, possibly because the patients with moderate-to-severe depression paid more attention to the intervention scheme and better adhered to the prescribed treatment.

A subgroup analysis of intervention theories showed that CBT-based interventions (including CBT alone and CBT combined with other theoretical interventions) significantly reduced depressive symptoms. However, we were unable to clarify which components worked best when the CBT was combined with other theories. A previous study [39] established that CBT-based interventions were more effective in improving the status of depression symptoms than educational interventions. Another review [41] uncovered that interpersonal psychotherapy (IPT) was more effective than other types of psychotherapies. Nonetheless, it is not clear why IPT is more effective than other therapies. A meta-analysis [42] also discerned that different types of psychotherapies (including CBT, IPT, PST, non-directed support therapy, and behavioral activation therapy) were effective in the treatment of adult depression, but no statistically significant difference was observed among the therapies. In our meta-analysis, we noted no significant difference between the groups, making it unsuitable for comparison between the groups. Further research is warranted to compare the advantages and disadvantages of interventions based on the theories established in this study.

In terms of intervention duration, it was found that the standardized mean difference (as Hedges’ g) was statistically significant in the t ≤ 6w, 6w<t ≤ 8w, and t>8w groups. It was also identified that the longer the intervention lasted, the better the improvement in depression. A previous study [43] has explored the difference between short-term and long-term psychotherapies in the treatment of depression. During the 5-year follow-up, it was noted that the short-term psychotherapy group recovered faster than the long-term psychotherapy group in the beginning. However, when the entire follow-up period was considered, the effect of the intervention on the long-term psychotherapy group was greater than that on the short-term psychotherapy group. This finding is consistent with the results of the present study. Because depression is prone to relapse, self-management needs to be continued for a long time to ensure that patients truly master the self-management skills and that they can apply them in their daily lives to achieve the best effect of the intervention.

In terms of intervention function, our subgroup analysis found that the group that communicated with the therapist displayed better improvements in the depressive symptoms than the group that did not communicate with the therapist and the group that communicated with the virtual healthcare provider. The results of a previous systematic review [44], consistent with the outcomes of this study, demonstrated that guided iCBT was associated with more effectiveness than unguided iCBT for individuals with depression. In general, web-based self-management programs without guidance were also effective for depression, although appropriate guidance from psychotherapists was believed to double the positive effects of the intervention. Some past studies have demonstrated that self-management with minimal therapist contact had positive effects on depression [28, 45]. Future research on web-based self-management of depression could be further explored in terms of the contact degree of therapists.

Regarding the status of adherence to the intervention, our results revealed that the intervention effect of the high adherence group was better, and the heterogeneity was small. However, there have been only 3 studies so far with adherence ≥90%; therefore, measures to improve treatment adherence should be considered in the research design of related studies in the future.

### Limitations

This meta-analysis has provided important information on the impact of web-based self-management interventions on depressive symptoms. However, certain limitations exist. First, this meta-analysis was heterogeneous. In this study, patients with mild, moderate, and severe depression were included and the interventions were based on various theories, which might have led to the high heterogeneity of the results. However, subgroup analysis was conducted for different conditions to explain the heterogeneity. Second, there were differences in the control group included in this meta-analysis. It included not only the waiting-list group but also the treatment-as-usual group and the online psychoeducation group. Nonetheless, subgroup analysis was carried out in this study. Third, it is difficult to perform further analysis. The studies have obvious design problems such as methodological flaws, high-risk allocation order, diverse measurement methods, lack of follow-up data, and difficulty in intervention control. Finally, psychotherapy is a long-term rehabilitation process, and the short-term effects may not be significant. The intervention time in this paper was short, and there was no long-term follow-up. Hence, further research is needed to clarify the long-term effects and the efficacy of the intervention.

## Conclusions

In summary, comprehensive evidence suggests that web-based self-management interventions can alleviate depressive symptoms. However, treatment via the internet is associated with challenges such as failure to ensure the patients’ adherence to the treatment, high rate of loss of follow-up, failure to determine the long-term effects of intervention, and the impact of individual depression severity on the effectiveness of the intervention. Future research is required to optimize and personalize web-based self-management interventions for depression.

As technology continues to advance and web-based self-management interventions for depression prove to be effective, the number of empirical studies in this field is growing rapidly. It could be stated that this kind of informational depression intervention is likely to be the future trend. Therefore, while continuing to design and evaluate the best web-based interventions, further research should be undertaken to develop viable approaches for implementing web-based interventions in healthcare systems.

## Supplementary Information

**Additional file 1.** Search strategy

**Additional file 2.** Subgroup analysis: Forest plot of intervention theory, time, adherence, and whether or not to communicate with the therapist.

**Additional file 3.** Publication bias (Egger's test and Trim-and-fill analysis).

## Data Availability

The data that support the findings of this study are available on request from the corresponding author. The data are not publicly available due to privacy or ethical restrictions.
