# Supplementary material for: Efficacy of web-based self-management interventions for depressive symptoms: a meta-analysis of randomized controlled trials
Source: BMC Psychiatry. 2021 Aug 11;21:398. doi: 10.1186/s12888-021-03396-8 (PMC8359554; doi:10.1186/s12888-021-03396-8)
Supplement: Supplementary file 1 — Additional file 1. Search strategy [file 12888_2021_3396_MOESM1_ESM.docx]

**Appendix 1**

**Search strategy**

**Pubmed**

(( "Depression/nursing"[Mesh] OR "Depression/prevention and control"[Mesh] OR "Depression/psychology"[Mesh] OR "Depression/therapy"[Mesh] OR “Depressive Symptoms” OR “Depressive Symptom” OR “Symptom, Depressive” OR “Symptoms, Depressive” OR “Emotional Depression” OR “Depression, Emotional” OR “Depressions, Emotional” OR “Emotional Depressions”) OR ( "Depressive Disorder/nursing"[Mesh] OR "Depressive Disorder/prevention and control"[Mesh] OR "Depressive Disorder/psychology"[Mesh] OR "Depressive Disorder/therapy"[Mesh] OR “Depressive Disorders” OR “Disorder, Depressive” OR “Disorders, Depressive” OR “Neurosis, Depressive” OR “Depressive Neuroses” OR “Depressive Neurosis” OR “Neuroses, Depressive” OR “Depression, Endogenous” OR “Depressions, Endogenous” OR “Endogenous Depression” OR “Endogenous Depressions” OR “Depressive Syndrome” OR “Depressive Syndromes” OR “Syndrome, Depressive” OR “Syndromes, Depressive” OR “Depression, Neurotic” OR “Depressions, Neurotic” OR “Neurotic Depression” OR “Neurotic Depressions” OR “Melancholia” OR “Melancholias” OR “Unipolar Depression” OR “Depression, Unipolar” OR “Depressions, Unipolar” OR “Unipolar Depressions) OR ( "Depressive Disorder, Major/nursing"[Mesh] OR "Depressive Disorder, Major/prevention and control"[Mesh] OR "Depressive Disorder, Major/psychology"[Mesh] OR "Depressive Disorder, Major/therapy"[Mesh] OR “Depressive Disorders, Major” OR “Major Depressive Disorders” OR “Major Depressive Disorder” OR “Paraphrenia, Involutional” OR “Involutional Paraphrenia” OR “Involutional Paraphrenias” OR “Paraphrenias, Involutional” OR “Psychosis, Involutional” OR “Involutional Psychoses” OR “Involutional Psychosis” OR “Psychoses, Involutional” OR “Depression, Involutional” OR “Involutional Depression” OR “Melancholia, Involutional” OR “Involutional Melancholia)) AND ( "Self-Management/economics"[Mesh] OR "Self-Management/education"[Mesh] OR "Self-Management/ethics"[Mesh] OR "Self-Management/methods"[Mesh] OR "Self-Management/organization and administration"[Mesh] OR "Self-Management/psychology"[Mesh] OR "Self-Management/standards"[Mesh] OR "Self-Management/statistics and numerical data"[Mesh] OR "Self-Management/trends"[Mesh] OR “Self Management” OR “Management, Self” )AND ("Randomized controlled trial" OR "random") AND ("Internet-Based Intervention"[Mesh] OR "internet-based" OR "web-based" OR ” internet-delivered” OR “internet” OR “online”)

**Web of science**

窗体顶端

| # 5 | #4 AND #3 AND #2 AND #1  *索引=SCI-EXPANDED, SSCI, A&HCI, CPCI-S, ESCI, CCR-EXPANDED, IC 时间跨度=所有年份* |
| --- | --- |
| # 4 | TS=(Internet-Based Intervention OR internet-based OR web-based OR internet-delivered OR internet OR online)  *索引=SCI-EXPANDED, SSCI, A&HCI, CPCI-S, ESCI, CCR-EXPANDED, IC 时间跨度=所有年份* |
| # 3 | TS=(Randomized controlled trial OR random)  *索引=SCI-EXPANDED, SSCI, A&HCI, CPCI-S, ESCI, CCR-EXPANDED, IC 时间跨度=所有年份* |
| # 2 | TS=(Self-Management OR Self Management OR Management, Self)  *索引=SCI-EXPANDED, SSCI, A&HCI, CPCI-S, ESCI, CCR-EXPANDED, IC 时间跨度=所有年份* |
| # 1 | TS=(Depression OR Depressive Symptoms OR Depressive Symptom OR Symptom, Depressive OR Symptoms, Depressive OR Emotional Depression OR Depression, Emotional OR Depressions, Emotional OR Emotional Depressions OR Depressive Disorder* OR Disorder, Depressive OR Disorders, Depressive OR Neurosis, Depressive OR Depressive Neuroses OR Depressive Neurosis OR Neuroses, Depressive OR Depression, Endogenous OR Depressions, Endogenous OR Endogenous Depression OR Endogenous Depressions OR Depressive Syndrome OR Depressive Syndromes OR Syndrome, Depressive OR Syndromes, Depressive OR Depression, Neurotic OR Depressions, Neurotic OR Neurotic Depression OR Neurotic Depressions OR Melancholia OR Melancholias OR Unipolar Depression OR Depression, Unipolar OR Depressions, Unipolar OR Unipolar Depressions OR Depressive Disorder, Major OR Depressive Disorders, Major OR Major Depressive Disorders OR Major Depressive Disorder OR Paraphrenia, Involutional OR Involutional Paraphrenia OR Involutional Paraphrenias OR Paraphrenias, Involutional OR Psychosis, Involutional OR Involutional Psychoses OR Involutional Psychosis OR Psychoses, Involutional OR Depression, Involutional OR Involutional Depression OR Melancholia, Involutional OR Involutional Melancholia)  *索引=SCI-EXPANDED, SSCI, A&HCI, CPCI-S, ESCI, CCR-EXPANDED, IC 时间跨度=所有年份* |

窗体底端

**Embase**

('depression'/exp OR 'central depression' OR 'clinical depression' OR 'depression' OR 'depressive disease' OR 'depressive disorder' OR 'depressive episode' OR 'depressive illness' OR 'depressive personality disorder' OR 'depressive state' OR 'depressive symptom' OR 'depressive syndrome' OR 'mental depression' OR 'parental depression') AND ('self care'/exp OR 'self care' OR 'self management' OR 'self treatment' OR 'self-management' OR 'self-nurturance' OR 'selfcare' OR 'selfmanagement' OR 'selftreatment') AND ('web-based intervention'/exp OR 'internet-based intervention' OR 'internet-intervention' OR 'online-based intervention' OR 'online-intervention' OR 'web intervention' OR 'web-based intervention') AND ('randomized controlled trial'/exp OR 'controlled trial, randomized' OR 'randomised controlled study' OR 'randomised controlled trial' OR 'randomized controlled study' OR 'randomized controlled trial' OR 'trial, randomized controlled')

**CINAHL**(MH "Depression/ED/NU/PC/RH/TH") AND ( (MH "Self-Management") OR (MH "Self Care") OR (MH "Personnel Management") ) AND ( randomized controlled trials or rtc or randomised control trials ) AND ( internet-based or web-based or internet based or web based )

**PsycINFO**
( depression or depressive disorder or depressive symptoms or major depressive disorder ) AND ( self-management or self-care or self-regulation or self-monitoring ) AND ( randomized controlled trials or rtc or randomised control trials ) AND ( "web-based intervention" OR "internet-based intervention" OR "internet-intervention" OR "online-based intervention" OR "online-intervention" OR "web intervention" OR "web-based intervention" )

**Cochorane**

( depression or depressive disorder or depressive symptoms or major depressive disorder ) AND ( self-management or self-care or self-regulation or self-monitoring ) AND ( randomized controlled trials or rtc or randomised control trials ) AND ( "web-based intervention" OR "internet-based intervention" OR "internet-intervention" OR "online-based intervention" OR "online-intervention" OR "web intervention" OR "web-based intervention" ) in Title Abstract Keyword - (Word variations have been searched)
