# Supplementary material for: Efficacy of web-based self-management interventions for depressive symptoms: a meta-analysis of randomized controlled trials
Source: BMC Psychiatry. 2021 Aug 11;21:398. doi: 10.1186/s12888-021-03396-8 (PMC8359554; doi:10.1186/s12888-021-03396-8)
Supplement: Supplementary file 2 — Additional file 2. Subgroup analysis: Forest plot of intervention theory, time, adherence, and whether or not to communicate with the therapist. [file 12888_2021_3396_MOESM2_ESM.docx]

**Appendix 2**

**Subgroup analysis: Forest plot of intervention theory, time, adherence, and whether or not to communicate with the therapist**

**
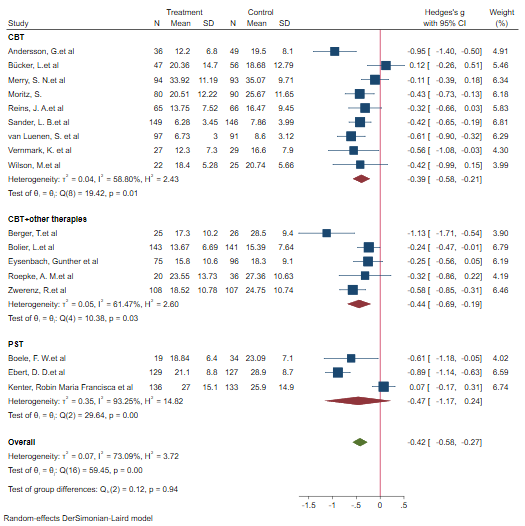
**

**Figure S1-A**

**
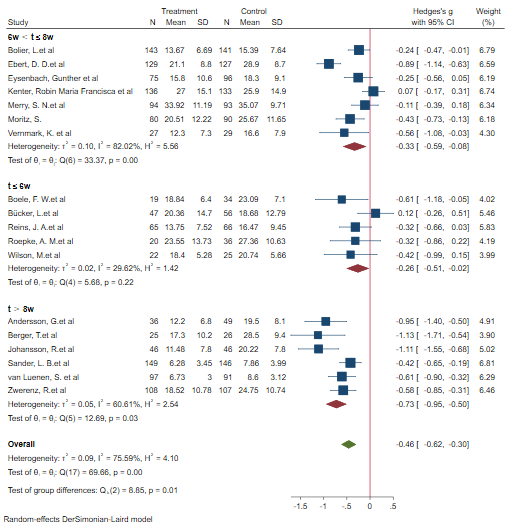
**

**Figure S1-B**

**
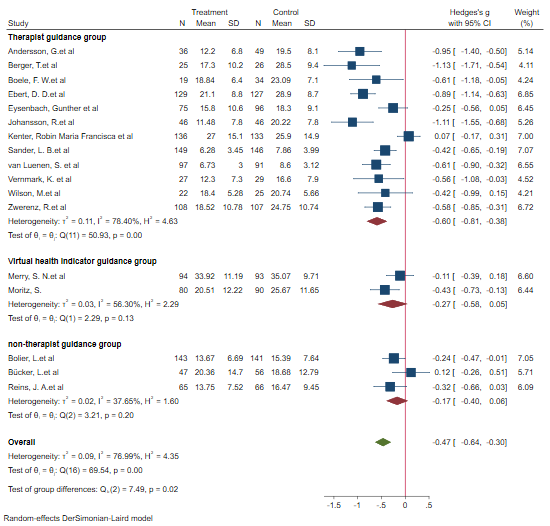
**

**Figure S1-C**

**
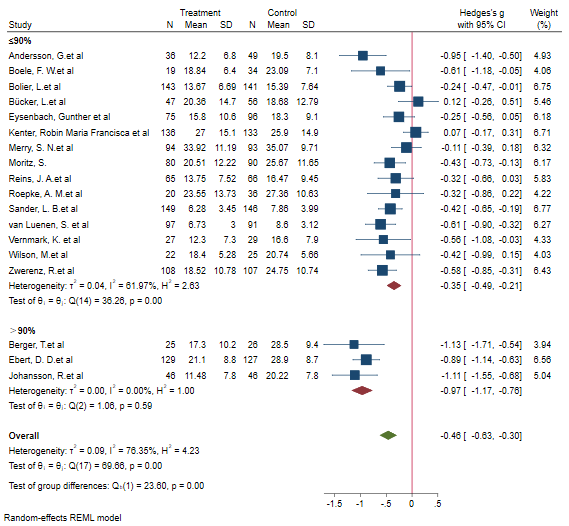
**

**Figure S1-D**
