# Supplementary figures and images for: Efficacy of web-based self-management interventions for depressive symptoms: a meta-analysis of randomized controlled trials
Source: BMC Psychiatry. 2021 Aug 11;21:398. doi: 10.1186/s12888-021-03396-8 (PMC8359554; doi:10.1186/s12888-021-03396-8)

Egger’s Test


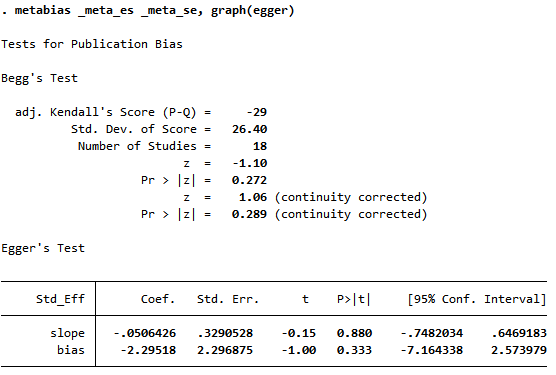


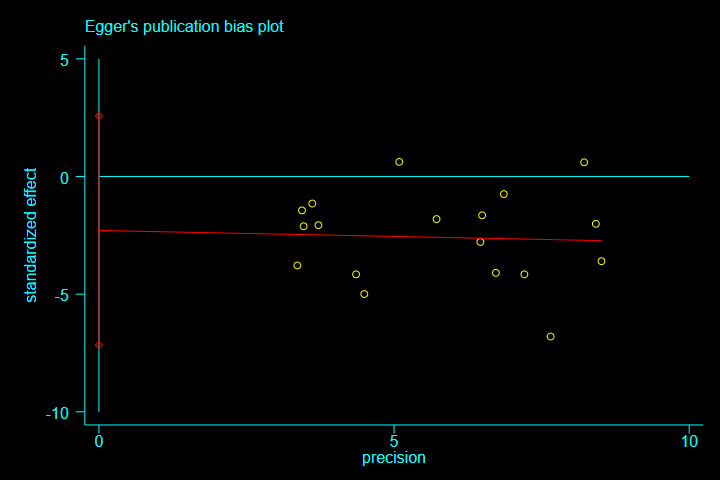


Trim-and-fill analysis


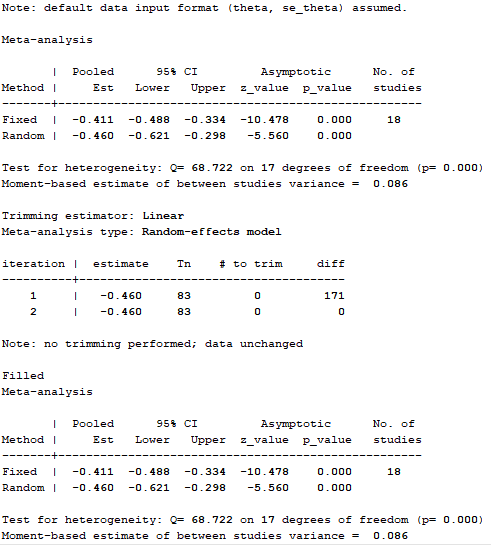


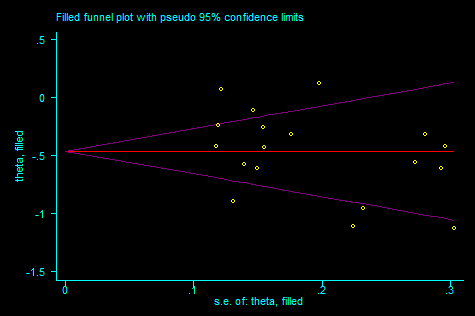

Supplement: Supplementary file 3 — Additional file 3. Publication bias (Egger's test and Trim-and-fill analysis). [file 12888_2021_3396_MOESM3_ESM.docx]
